# Incidence of bullous pemphigoid in Sweden 2005–2012: a nationwide population-based cohort study of 3761 patients

**DOI:** 10.1007/s00403-017-1778-4

**Published:** 2017-09-05

**Authors:** Kristofer Thorslund, Oliver Seifert, Karin Nilzén, Carina Grönhagen

**Affiliations:** 1 Dermatology and Venereology Unit, Department of Medicine Solna, Karolinska Institutet, Stockholm, Sweden; 2 Division of Dermatology and Venereology, Region Jönköping County, Jönköping, Sweden; 3 Department of Clinical and Experimental Medicine, Faculty of Health Sciences, Linköping University, 581 83 Linköping, Sweden; 4 Department of Dermatology, Skåne University Hospital, Lund University, Malmö, Sweden

**Keywords:** Incidence, Bullous pemphigoid, Population-based cohort study

## Abstract

Studies that report the incidence of bullous pemphigoid from validated nationwide population-based registries are rare. The aim of this study was to estimate the incidence of bullous pemphigoid in Sweden 2005–2012. A population-based open cohort study was designed including all patients diagnosed by a dermatologist with bullous pemphigoid (BP) in Sweden from 2005 to 2012 (*n* = 3761), identified from the National Patient Register (NPR). The diagnosis of bullous pemphigoid in the NPR was recently validated from medical records, histopathological and immunopathological data by our group in a previous study. The average annual incidence of bullous pemphigoid was 7.1/100,000 (95% CI 6.5–7.7). Female to male ratio was 1.2:1, mean age at diagnosis was 78.9 years. The age-specific incidence rate increased markedly after 80 years of age with an incidence peak between 90 and 99 years of age, 81.9/100,000 (95% CI 75.0–89.2). This large nationwide cohort study presents an adjusted incidence of BP of 7.1/100,000 (95% CI 6.5–7.7) in Sweden. The incidence of bullous pemphigoid is higher than expected and bullous pemphigoid is a common disease in the elderly population.

## Introduction

Autoimmune bullous diseases are a group of chronic, blistering diseases of the skin, the most common of which is bullous pemphigoid (BP). BP is a severe skin disease that causes much suffering and significantly impacts morbidity of those affected. BP is associated with autoantibodies to different structures in the skin, resulting in skin losses and subepidermal blisters. In BP the autoantibodies are directed against components of the hemidesmosomes, BP-antigen 180 and BP-antigen 230 [25].

Knowledge about the incidence of BP is still limited and most studies are hampered by a retrospective design and a small number of included BP patients (*n* = 27–140) [2–4, 10, 15, 18, 22, 23, 26, 29, 31–33]. BP is known to have differing incidence rates in diverse parts of the world. Of importance, annual incidences from European studies vary between 0.3 and 4.3/100,000 [1]. A recent prospective study from Switzerland, including 140 BP patients, estimated the incidence to 1.2 cases/100,000 inhabitants/year [22]. The corresponding numbers in Germany, France, Finland and USA are 1.3, 2.1, 1.4 and 2.4/100,000, respectively [4, 5, 12, 17]. In a large study (*n* = 869) from the UK, where data from primary care was used, the incidence rate was higher, 4.3/100,000 [19].

BP most typically occurs in the elderly, usually in individuals in their late 70’s. Several European countries have noticed an increasing incidence in the last decade which could be attributed to an aging population and the use of improved diagnostic tools [12, 30]. Since average life expectancy is on the increase in Europe, there is good reason to believe that BP will also continue to increase. The incidence of BP in Sweden remains sparse, as very few reliable studies have assessed the overall age- and gender-specific distribution of BP’s incidence. The aim of this study was to estimate the age- and gender-specific incidence rates of BP throughout the whole of Sweden using a validated population-based registry.

## Materials and methods

### Study design

This nationwide cohort study has included all patients in Sweden (above 20 years of age) diagnosed with BP by a dermatologist [International Classification of Diseases (ICD) code, ICD-10: L12.0, L12.0A, L12.0B, L12.0W, L12.8 and L12.9] in the National Patient Register (NPR) for the first time between 2005 and 2012. The diagnosis of BP in the NPR has recently been validated by us through a standardized review of medical records, and showed a positive predictive value of 92% [14]. Only patients who fulfilled clinical, histopathological and immunological criteria for BP were included. The NPR includes both specialized outpatient and inpatient care. The outpatient register started in 2001 and a pool of prevalent, preexisting cases was registered for the first time over the first few years (2001–2004). New registrations subsequently stabilized at a lower number of cases, assumed to represent the annual incidence average [16]. Thus, patients diagnosed with BP for the first time from 2005 onwards were regarded as incident cases, implying that they did not have a diagnosis of BP in at least the four preceding years. The unique personal identification number (PIN) assigned to all residents in Sweden and recorded in all health registries was used to eliminate any risk of duplication.

We excluded residents under the age of 20 since BP is extremely rare in this age group. When estimating age- and gender-specific incidence rate, the entire Swedish population above 20 years of age 2005–2012 (*n* = 7,122,447) was assumed to be at risk. Such incidence rates were calculated by dividing the number of new cases in each age and gender group by the age- and gender- and calendar year-specific population figures obtained from Statistics Sweden (http://www.scb.se). To estimate the incidence rate we divided the average number of cases (*n* = 470) diagnosed with BP during 2005–2012 by the mean of the whole background population in Sweden (>20 years) during the corresponding years (*n* = 7,122,447).

### National Patient Register

The Swedish National Patient Register was launched by the National Board of Health and Welfare in 1964 and has virtually complete coverage of all inpatient care—both public and private—since 1987 in Sweden. The reporting is mandatory and from 2001 data from specialized outpatient care is also included, although primary care is not [27]. Inpatient coverage is close to 100% [21] and outpatient coverage is 87% with both public and private caregivers being reported in the register [28].

From 1997 and onwards, diagnoses continue to be coded according to the ICD 10th revision (ICD-10). The NPR includes information about patient characteristics (PIN, sex, age and county of residence), administrative data, hospital identification and medical data from major interventions and discharge diagnoses—one main and up to seven secondary discharge diagnoses—as well as diagnostic dates.

### Sensitivity analysis

To further improve the accuracy of this incidence estimate, a sensitivity analysis was performed. The specific diagnosis of BP was recently validated in the NPR in two Swedish counties and was shown to have high validity, with a positive predictive value of 92% [14]. Parallel to the validation, all cases diagnosed with BP according to the Pathology Registry (SNOMED codes D3618 and D36180) in those two counties, 2005–2012, were also retrieved. There was only one pathology laboratory in each county. All retrieved cases from the Pathology Registry were matched against the BP cases found in the NPR. Cases found only in the Pathology Registry were manually retrieved and both immuno- and histopathology data and medical records were reviewed by a dermatopathologist and subsequently confirmed to be BP. Such cases were then regarded as “missing incident cases of BP” in the NPR. The result from these two counties was then statistically generalized over the entire country in the sensitivity analysis.

### Ethics

The Regional Ethical Review Board in Stockholm, Sweden approved this study.

### Statistics

Student’s *t* test for parametric data and the Chi-square test for non-parametric data were used when comparing means between groups. Confidence intervals for estimated incidences were calculated assuming Poisson distributed number of newly diagnosed patients. All the data were analyzed using the statistical package STATA^®^ Statistical Software (release 11.1; Stata-Corp, College Station, TX, USA) or Microsoft Excel^®^. Differences were considered to be significant at *p* < 0.05.

## Results

We found 3761 incident cases of BP in Sweden between 2005 and 2012. Mean age at diagnosis was 78.9 years (SD 12.1, range 20–102) and 54% were females. There was no statistically significant difference in the age at diagnosis between men and women. 96.7% (*n* = 3636) of all BP patients were 50 years or older at the time of BP diagnosis, 81.2% (*n* = 3053) were 70 years or older and 54.8% (*n* = 2062) were 80 years or older. The majority were diagnosed in outpatient care (81.2%, *n* = 3055) and had been given a main diagnosis of BP (82.3%, *n* = 3096).

**Fig. 1:**
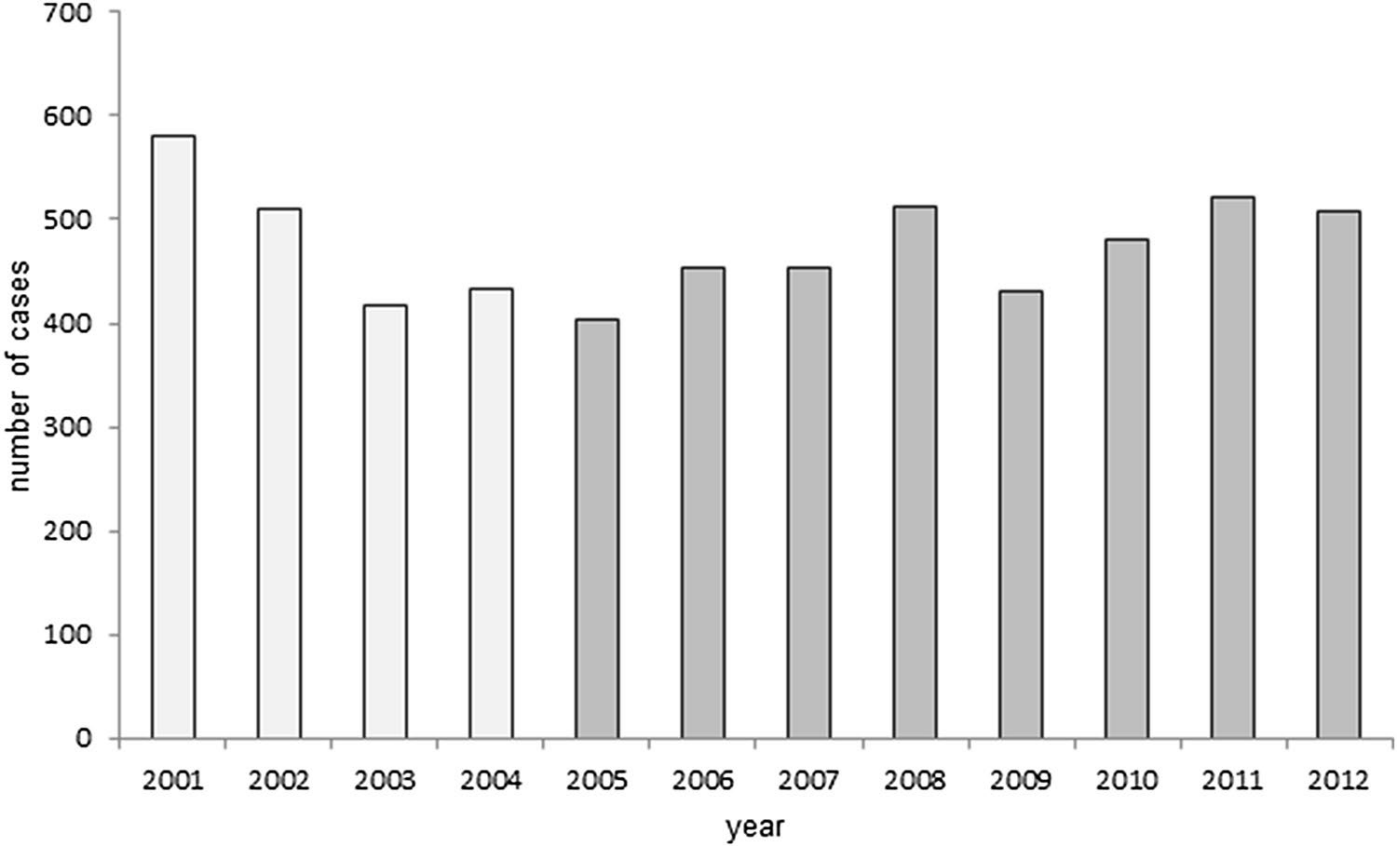
Incident and prevalent cases of bullous pemphigoid (BP) 2001–2012 in Sweden. Number of cases diagnosed with BP in Sweden, 2001–2012, from the National Patient Register (NPR)—both inpatient and outpatient care. Patients regarded as incident cases were above 20 years of age and diagnosed with BP for the first time in 2005 (dark colored bars). To estimate the incidence rate for the past 8 years (2005–2012), we divided the average annual number of cases (*n* = 470) diagnosed with BP during 2005–2012 by the whole background population in Sweden (>20 years) during the corresponding years (*n* = 7,122,447). Incidence of BP in Sweden 2005–2012 for residents above 20 years of age according to data from the NPR = 470/7,122,447 = 6.6 cases/100,000 (95% CI 6.0–7.2)

From 2005 to 2012, the annual number of BP cases fluctuated (ranging from 403 to 522 per year); however, there was no perceivable trend towards either increasing or decreasing numbers. The average annual incidence rate of BP for residents above 20 years of age was 6.6/100,000 (95% CI 6.0–7.2) (Fig. 1).

**Fig. 2:**
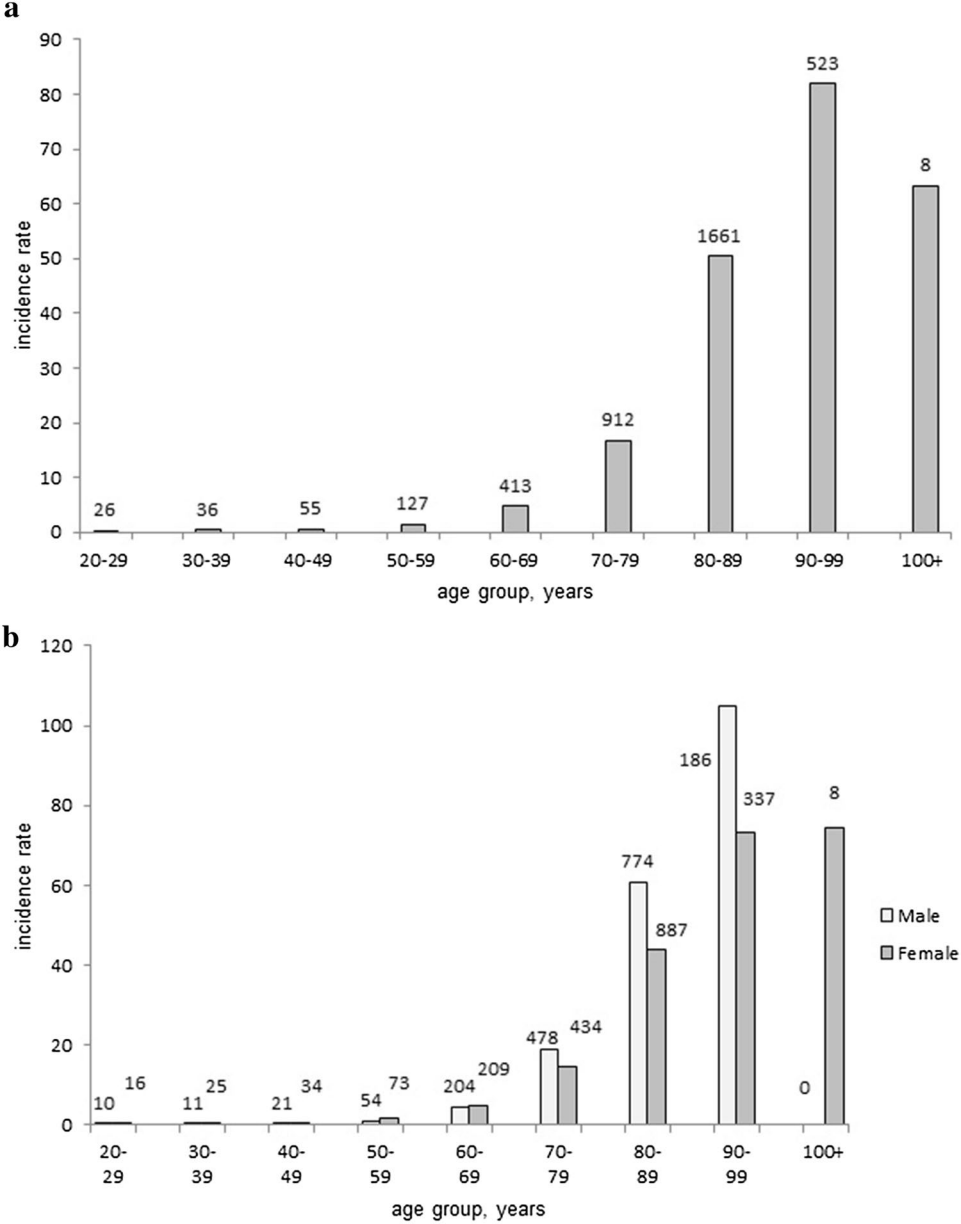
**a** Age-specific incidence rate (IR) of BP from 2005 to 2012 in Sweden. Average age-specific IR of bullous pemphigoid (BP), adjusted for the Swedish population in 2005–2012 per 100,000 people. The number above the bars is the actual number of patients within each age group on which the incidence estimate is based. **b** Age- and gender-specific incidence rate (IR) of BP from 2005 to 2012 in Sweden. Average age- and gender-specific incidence rate of bullous pemphigoid and its subsets (adjusted to the Swedish population in 2005–2012 per 100,000 inhabitants). The number above the bars is the actual number of patients within each age and gender group on which the incidence estimate is based

The age and gender-specific incidence rates are presented in Fig. 2. It shows a marked increase after 80 years of age with an incidence peak between 90 and 99 years of age, 81.9/100 000 (95% CI 75.0–89.2) (Fig. 2a). BP is uncommon in people under 50 years of age (incidence 0.4/100,000).

Men had a significantly higher incidence compared to women from 70 years of age onward, with the greatest difference being between 90 and 99 years of age: 104.8/100,000 (95% CI 90.1–121.1) for men and 73.1/100,000 (95% CI 65.5–81.3) for women, *P* < 0.001 (Fig. 2b). There were few (*n* = 8) patients 100 years and older and all of them were female.

In addition, a sensitivity analysis was performed (Table 1). The Pathology registries in two counties were also checked for a diagnosis of BP. Eleven BP cases were never registered in the NPR, which suggests there is an underestimation of incident cases in the NPR of about 3.6%. Previous validation of BP in the NPR through a standardized review of medical records showed a positive predictive value of 92% [14]. The adjusted incidence rate of BP is 7.1/100,000 (95% CI 6.5–7.7).

**Table 1:** Sensitivity analysis of the patients with bullous pemphigoid (BP) according to histopathological records but not identified in the National Patient Register (NPR)

Sensitivity analysis
11 cases of BP were only found in the Pathology Register^a^ and not coded for in the NPR implying an underestimation of about 3.6% (11/307) in the NPR when compared with the Pathology Register
Outpatient care coverage 87%; 81.2% of the BP cases were diagnosed in outpatient care Inpatient care coverage 100%; 18.8% of the BP cases were diagnosed in inpatient care
Average number of BP cases identified annually in the NPR 2005–20012: 470
Estimated no. of BP patients if full coverage of both in- and outpatient care: ((470 × 0.812)/0.87) + ((470 × 0.188)/1) = 527
There is an underestimation of about 3.6% in the NPR: 527/0.964 = 547
Positive predictive value for the NPR was 92% (283/307) when medical records from two counties were reviewed^b^
The estimated no. of true BP patients: 547 × 0.92 = 503
The adjusted incidence of BP: 503/7,122,447 = 7.1/100,000 (95% CI 6.5–7.7)

^a^ These BP cases were also validated against medical records

^b^ Grönhagen et al. [14]

## Discussion

From this large population-based cohort study, we estimated adjusted annual incidence rates of BP in Sweden to be 7.1/100,000 inhabitants. This estimate was based on the nationwide NPR, which had previously been validated for the specific diagnosis of BP [14]. The overall incidence increased with age and reached its maximum between the ages of 90 and 99 years (81.9/100,000). Men had a significantly higher incidence rate compared to women from 70 years of age, which confirmed findings in other studies and can be explained by fewer men reaching an advanced age [15, 18, 19].

A retrospective study from the UK found an incidence rate of 4.3/100,000 while most other studies found even lower incidence rates [2–4, 10, 15, 22, 23, 26]. It is difficult to compare these studies since they had different designs. The present study included nationwide prospectively collected data and 3761 BP cases were identified. Another reason for the higher incidence rate in our study is that only individuals above 20 years of age were included, whereas other studies calculated incidence rates on the basis of the entire population even though BP is known to be extremely rare during childhood.

BP is less common in people under 50 years of age and the present study found an incidence of 0.4/100,000 in this age group, which is in line with a previous study [22]. Regarding individuals above age 80 our data showed higher BP incidence (50–82/100,000) compared with previous studies (19–50/100,000) [4, 17, 18]. The age- and gender-specific incidence rates in the present study were calculated based on the corresponding age and gender distribution of the entire Swedish population, to enhance accuracy. Mean age at diagnosis (78.9 years) in the present study was in line with previous study results of 80–82 years [17, 19] and 74–76 years [3, 9, 10, 22, 33].

BP has previously been reported to be slightly more frequent among females (1.5:1 and 1.3:1, respectively) [17, 22]. This study found a female to male ratio of 1.2:1 when adjusted to the gender distribution in the general population, which is in line with another study from Taiwan [8]. The gender distribution in the general population must be taken into account since BP is more frequent in the elderly, and women are overrepresented in the older age groups.

The present study has a number of significant strengths. The NPR is a nationwide register that includes the entire Swedish population; the data on inpatient care have nearly 100% coverage and that on outpatient care has coverage of 87%. The internal validity of the NPR is robust with <1% missing data [21]. The specific diagnosis of BP has recently been validated in the NPR and found to have a high positive predictive value, ensuring a high level of accuracy [14]. To further improve the accuracy of the incidence estimates, a sensitivity analysis was performed. This led to an increase in the incidence rate by 8% in the two counties where the validation was performed. These findings were then generalized to cover the whole of Sweden. The data from the NPR were collected in a prospective way even though the information used in this study was withdrawn retrospectively. As this study was nationwide and lasted 8 years, selection recruitment bias was limited as were the possible seasonal and temporal variations in the BP incidence.

One limitation of this study is that we did not capture BP that did not lead to a consultation with a doctor; still, we believe there were very few cases where patients developed blisters and/or severe itching without seeking medical advice. BP cases that were misdiagnosed as something else clinically and never underwent histopathological testing would also not have been captured.

Primary care is not included in the NPR. However, those cases handled in primary care, where a skin biopsy was taken that showed consistency with BP, were captured due to the Pathology Registry incorporated in this study.

BP is a severe skin disease with massive impact on quality of life and morbidity, and increasing incidence rates implies heightened risk for the growing elderly population. Care givers and national health care systems need to pay close attention to meeting the demands posed by an increasing patient number.

The reason for the increased incidence of BP with age is unknown. Although in a majority of BP cases no specific triggering factor can be identified, such factors as the increased use of drugs by the elderly—sometimes several drugs simultaneously—might explain for the steep increase in BP after 80 years of age. Drugs reported to cause BP include diuretics, analgesics and antibiotics [20], all frequently used in the elderly population. BP is also associated with various neurological diseases such as dementia and Parkinson’s disease [8], which are also more common in the elderly. Another potential cause for the increased BP incidence with age could be related to loss of self-tolerance in the immune system [22].

Autoimmune diseases such as multiple sclerosis and diabetes mellitus type I [13, 24] are known to be more prevalent in the Scandinavian countries than elsewhere, and the same may apply to BP. This could be explained by phenotypic predisposition in Scandinavia but overexposure to UV radiation has also been suggested as a risk factor for BP since the Scandinavian population is known to have a high UV exposure [6, 7, 11].

In conclusion, this is the first nationwide population-based registry study to present valid and robust data on the incidence of BP in an entire country. In total 3761 BP cases were included and the adjusted incidence rate was estimated to 7.1/100,000. This is the highest incidence level in Europe, thus confirming BP as a relatively common disease in the elderly population. Future studies may be able to reveal more information about the mechanisms resulting in BP and further explain its higher incidence rate in Sweden.
